# Defining late onset occult asymptomatic cardiotoxicity in childhood cancer survivors exposed to anthracycline therapy: a cardiac magnetic resonance imaging study

**DOI:** 10.1186/1532-429X-15-S1-P163

**Published:** 2013-01-30

**Authors:** Olga H Toro-Salazar, Kan N Hor, Michael O'Loughlin, Georgine Burke, Jeff A Stainsby, Eileen Gillan, Bruce T Liang, Michael Taylor

**Affiliations:** 1 Cardiology, Connecticut Children's Medical Center, Hartford, CT, USA; 2 Cardiology, Cincinnati Children's Hospital Medical Center, Cincinnati, OH, USA; 3 Radiology, Hartford Hospital, Hartford, CT, USA; 4 Research, Connecticut Children's Medical Center, Hartford, CT, USA; 5 Cardiology, University of Connecticut Health Center, Farmington, CT, USA; 6 Cardiac & Interventional Applications, GE Healthcare, Toronto, ON, Canada; 7 Hematology/Oncology, Connecticut Children's Medical Center, Hartford, CT, USA; 8 Pediatric, University of Connecticut, Farmington, CT, USA

## Background

There are over 270,000 childhood cancer survivors in the US. Of these survivors, more than 50% have been treated with anthracyclines and are at risk of developing progressive cardiotoxicity. Novel cardiac magnetic resonance imaging (CMRI) techniques are now able to reliably detect diffuse myocardial fibrosis and changes in regional myocardial function. We hypothesized that these novel CMRI techniques will identify occult asymptomatic cardiotoxicity in a cohort of childhood cancer survivors with normal global systolic function.

## Methods

Twenty seven long-term childhood cancer survivors between 11.8-28.8 years with a cumulative dose >240mg/m2 (mean 363±89) and normal systolic function (SF>29%) were studied 2.4-24 years after exposure to anthracycline therapy. Patients underwent CMRI techniques to characterize changes in T1 relaxation time, left ventricular myocardial peak circumferential and longitudinal strain parameters and were analyzed using the 17-segment model. Extracellular volume (ECV) was measured in 13 subjects all of whom were late gadolinium enhancement (LGE) negative. We performed standard CMRI assessment and quantification of myocardial mass, end-systolic and end-diastolic volumes, ejection fraction, and end systolic fiber stress.

## Results

**Figure 1:**
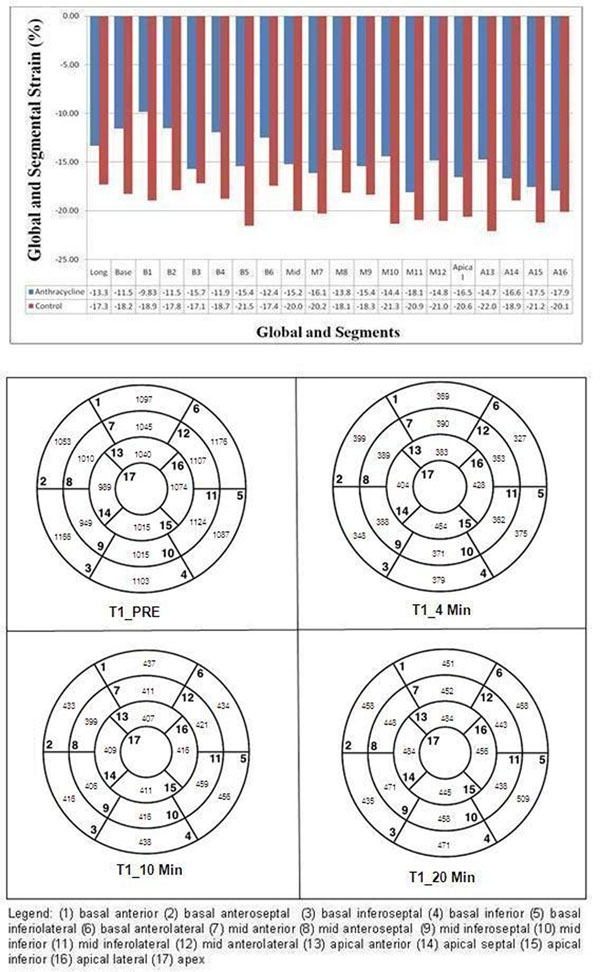


**Figure 2:**
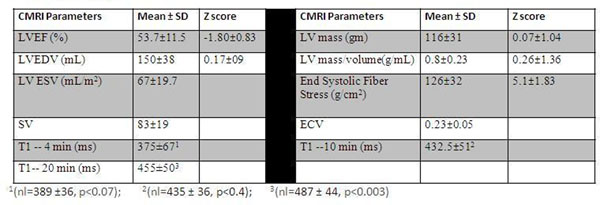
CMRI parameters

Twenty seven of 60 planned subjects have been imaged. End systolic fiber stress was significantly increased with higher cumulative anthracycline dose (R2=0.18, p<0.03) and younger age at diagnosis (R2=0.20, p<0.02). Lower average circumferential strain magnitude (εcc) and regional changes in peak circumferential strain were seen in multiple segments despite normal values of global systolic function by echocardiography and CMRI (Figure1). T1 maps are depicted in Figure 1. The mean T1 values of the myocardium were not significantly different between patients and controls at 4 min (375±67ms vs.389±36, p<0.07) and 10 min (433±52 ms vs.435±36, p<0.39), but were significantly lower at 20 minutes (455±50ms vs. 487±44, p<0.003) (Figure 2). Low myocardial T1 at 20 minutes was significantly associated with increases in end systolic fiber stress (R2=0.7, p<0.002). Higher mean ECV was observed in patients with cumulative dose ≥400mg/m2 (0.27 vs. 0.21, p<0.05).

## Conclusions

In this study we demonstrate that changes in T1 mapping-derived relaxation time and left ventricular myocardial peak circumference strain are present in asymptomatic post-chemotherapy childhood patients who have normal standard CMRI parameters. Circumferential strain analysis and measurement of the T1 myocardial relaxation time by CMRI may accurately identify occult cardiovascular dysfunction in patients exposed to high dose anthracyclines. Thus, this may aid in the evaluation of therapies aimed at reducing adverse cardiac remodeling and preventing heart failure in childhood cancer survivors exposed to anthracyclines.

## Funding

St. Baldrick's Foundation Supportive Care Research Grant

